# The Next Infodemic: Abortion Misinformation

**DOI:** 10.2196/42582

**Published:** 2023-05-04

**Authors:** Sherry L Pagoto, Lindsay Palmer, Nate Horwitz-Willis

**Affiliations:** 1 UConn Center for mHealth and Social Media UConn Institute for Collaboration in Health, Interventions, and Policy University of Connecticut Storrs, CT United States; 2 Department of Allied Health Sciences University of Connecticut Storrs, CT United States; 3 Planned Parenthood Advocacy Fund of Massachusetts Boston, MA United States

**Keywords:** abortion, reproductive health, misinformation, online, infodemic, misleading information, online health information, health authority, public health, abortion, women's health

## Abstract

The World Health Organization (WHO) defines an infodemic as the proliferation of false or misleading information that leads to confusion, mistrust in health authorities, and the rejection of public health recommendations. The devastating impacts of an infodemic on public health were felt during the COVID-19 pandemic. We are now on the precipice of another infodemic, this one regarding abortion. On June 24, 2022, the Supreme Court of the United States (SCOTUS) decision in *Dobbs v. Jackson Women’s Health Organization* resulted in the reversal of *Roe v. Wade*, which had protected a woman’s right to have an abortion for nearly 50 years. The reversal of Roe v. Wade has given way to an abortion infodemic that is being exacerbated by a confusing and rapidly changing legislative landscape, the proliferation of abortion disinformants on the web, lax efforts by social media companies to abate abortion misinformation, and proposed legislation that threatens to prohibit the distribution of evidence-based abortion information. The abortion infodemic threatens to worsen the detrimental effects of the *Roe v. Wade* reversal on maternal morbidity and mortality. It also comes with unique barriers to traditional abatement efforts. In this piece, we lay out these challenges and urgently call for a public health research agenda on the abortion infodemic to stimulate the development of evidence-based public health efforts to mitigate the impact of misinformation on the increased maternal morbidity and mortality that is expected to result from abortion restrictions, particularly among marginalized populations.

The World Health Organization (WHO) defines an infodemic as the proliferation of false or misleading information that leads to confusion, health risk behaviors, mistrust in health authorities, and the rejection of public health recommendations, all of which exacerbate a public health crisis [1]. False or misleading information can be classified as either misinformation or disinformation [2]. Misinformation refers to information that is false and being shared by someone who believes it to be true, whereas disinformation is information that is false and being shared by someone who is aware that it is false but intends to deceive others. The COVID-19 pandemic illuminated the catastrophic effects of an infodemic, perhaps more than any public health event that preceded it. We are now on the precipice of another infodemic, this one regarding abortion.

Approximately 20% of women in the United States have had an abortion [3]. On June 24, 2022, the Supreme Court of the United States (SCOTUS) decision in *Dobbs v. Jackson Women’s Health Organization* allowed a Mississippi law that proposed to make abortion illegal after 15 weeks to stand, which effectively overturned *Roe v. Wade* [4], a 1973 SCOTUS decision that affirmed the right to have an abortion under the 14th amendment [5]. With the reversal of *Roe v. Wade*, a perfect storm of factors is driving an abortion infodemic including a confusing and rapidly changing legislative landscape [6], the proliferation of abortion disinformants [7], lax efforts by internet and social media companies to abate abortion misinformation [8], proposed legislation and policies that prohibit the provision of accurate information about abortion [9-13], and deep stigma around publicly discussing abortion felt by both patients and providers [14]. The abortion infodemic threatens to exacerbate the detrimental effects that are expected following the *Roe v. Wade* reversal on maternal morbidity and mortality [15]. Specifically, a recent study estimated that eliminating abortion for 1 year would increase maternal mortality by 24% overall and 39% for Black women, simply because pregnancy has a higher fatality rate than abortion [16], for which death is extremely rare [17]. Abortion restrictions increase maternal morbidity and mortality by forcing women to carry out pregnancies that pose risks to their health, forcing patients with cancer to delay cancer care until after pregnancy [18], and restricting how physicians can treat miscarriages [19]. Because pregnancy poses serious morbidity and mortality risks, the dual rights to bodily autonomy *and* medically accurate information are paramount to protect pregnant patients who must weigh their personal risk in the decision to carry out a pregnancy.

Infodemics, by producing a high volume of misinformation that travels quickly on the internet and social media, obstruct a patient’s ability to find medically accurate information to inform their health care decisions, and this can have dire consequences. In the case of abortion, patients may conduct a self-managed abortion based on misinformation on the internet about “home remedy” abortions (eg, using insect repellent or illicit drugs) [20]. Misinformation about abortion can also exacerbate maternal mortality when it influences policy makers who then use it to justify abortion bans, given that the bans themselves are expected to increase the national maternal mortality rate. For example, Ohio legislators, operating on misinformation, put forth a bill that would prohibit physicians from terminating ectopic pregnancies but instead requiring them to “reimplant” the embryo in the uterus, which is medically impossible [21]. Accessible and accurate information about abortion is essential for patients, policy makers, and the general public. In this piece, we urgently call for a public health research agenda on the abortion infodemic to (1) stimulate the development of evidence-based public health efforts to mitigate the negative impacts of misinformation on patients, health care providers, and policy makers; (2) build better systems for providing medically accurate information to pregnant patients to inform their health care decisions; and (3) educate the public on the consequences of abortion bans on maternal morbidity and mortality [22].

Abortion misinformation is not new. For decades, it has proliferated, often for the purpose of fueling the antiabortion agenda [23]. A 2014 study of “crisis pregnancy center” websites found that 80% contained misinformation, typically perpetuating myths about abortion-related health risks [24]. A subsequent study of the top 5 web pages on abortion medication produced by Google searches found that most contained similar types of misinformation [25]. Specifically, top web pages stated that abortion medication can cause mental illness, negatively impact infertility, and increase risk for mortality. Misinformation affects individuals regardless of their stance on abortion. One survey found that 67% of respondents identifying as “pro-choice” and 88% of respondents identifying as “pro-life” said that giving birth is safer or just as safe as getting an abortion [26]. In fact, childbirth has a mortality rate that is 50- to 130-fold greater than that of abortion [27]. Research is needed to classify the types of misinformation circulating on the web—and on social media specifically—and the impact of misinformation exposure on health care decisions.

The reversal of *Roe v. Wade* was immediately followed by a steep spike in web-based searches for information about abortion, which, combined with the finding that top Google hits for abortion medication are for websites that contain misinformation [25], means reproductive health misinformation is highly accessible—an indicator of an infodemic. For instance, 72 hours after the SCOTUS draft opinion leak on Dobbs v. Jackson Women’s Health Organization in June 2022, medication abortion Google searches were 162% higher than typical for this time frame and were especially high in states with restrictive abortion laws [28]. People who have the least access to abortion are the most likely to encounter medical misinformation, and this could lead to delayed or inappropriate care, which has implications for maternal morbidity and mortality [29]. Since the reversal of *Roe v. Wade*, popular press outlets have reported on social media trends promoting the use of dangerous or ineffective abortion procedures including herbs, exercise, home vacuum aspirations, insect repellent, essential oils, illicit drugs, alcohol, birth control pills, self-injury, caffeine pills, and vitamin C [20,30]. Misinformation also abounds about abortion causing fetal pain [31], infertility, death, life-threatening complications, and mental illness [32]. Further, modern misinformants perpetuate dangerous antiquated myths that abortion is never used to save a pregnant person’s life; that abortions can be reversed [33]; that infants are born alive during abortions [34]; and that abortion is promoted for human sacrifice, eugenics, or to further a scientific agenda [31]. Abortion misinformation in Spanish targeting Latinas has also been observed recently, including messaging that advises women that abortion is illegal in places where it is legal [35,36]. At present, there are no coordinated efforts to document or combat abortion misinformation.

The abortion infodemic has several unique challenges relative to other infodemics, and these have serious implications for public health efforts. One such challenge is that providing medically accurate abortion information in states with vigilante laws such as Texas and Oklahoma may subject one to litigation if authorities consider communications to have “aided or abetted” an abortion [37]. Going further, South Carolina legislators proposed a bill that would outlaw the provision of information about how to obtain an abortion via websites or telephone [38]. In Mississippi, the attorney general subpoenaed a health education nonprofit organization that put up billboards pointing women to a website about abortion medication [39]. All of these efforts to stifle information are consistent with the National Right to Life Committee blueprint, which recommends outlawing “giving instructions over the telephone, internet, or any other medium” or “hosting or maintaining a website, or providing internet service, that encourages or facilitates” abortion [13]. Given efforts to punish the provision of accurate health information regarding abortion, common approaches to mitigating misinformation established during the pandemic, including leveraging health care providers, community leaders, and public health campaigns [40], may entail legal risks or at the very least intimidate individuals from engaging in such efforts. A second challenge to addressing the abortion infodemic is that health care providers may be reticent to educate patients and the public about abortion, fearing personal and professional repercussions. Indeed, outspoken physicians have become targets for harassment [41], as has also been the case in the COVID-19 pandemic [42], but even worse, physicians providing abortion have been murdered by abortion opponents [43]. These chilling events can have a silencing effect among health care providers, which may reduce the amount of medically accurate information they are willing to share with patients or on the internet. A third unique challenge of the abortion infodemic is how misinformation not only affects the public but also health care professionals and administrators to the extent that abortion legislation is vaguely written and left open to interpretation [27]. Confusion is a key ingredient of infodemics. The Texas Medical Association reported that some hospitals are requiring physicians to turn away pregnant patients with complications due to the fear of litigation in the event that complications are considered to have not met the unclear legal threshold for “life-threatening” [44]. To the extent that physicians, fearing litigation, err on the side of withholding or delaying care, the deleterious effects of abortion restrictions on maternal morbidity and mortality will be compounded [15]. Relatedly, a fourth challenge is that medical training in abortion is constrained in states with severe abortion restrictions [45]. This may result in a workforce that lacks skills and knowledge in early pregnancy management. In fact, nearly half of obstetrics and gynecology residents are currently in states that have banned or severely restricted abortion care, which means that a substantial proportion of the workforce may lack appropriate training and experience to provide the highest quality of care to patients for whom a pregnancy must be terminated [46]. Health care providers are a trusted source of health information for many people [47]; however, to the extent that medical training is compromised by abortion restrictions, health care providers may then be compromised in their ability to provide medically accurate information and health care, and this in turn may impact patient trust.

A troubling paucity of research exists about abortion misinformation as evidenced by a 2021 review of the literature that identified only 9 studies [23]. In 2020, the WHO responded to the COVID-19 infodemic by developing a framework for health authorities on managing the infodemic, facilitating an international agreement to improve access to accurate COVID-19 information signed by 132 countries, and providing guidelines for a public health research agenda that included trainings and conferences [1]. The abortion infodemic requires similar efforts in the United States, including partnerships with other nations facing attacks on reproductive rights and poor access to accurate information regarding maternal health broadly. Such a research agenda will require transdisciplinary teams to address the following priorities: surveillance, impacts and health outcomes, health communication interventions, contextual factors influencing intervention efficacy, and preventive measures. Specifically, epidemiology and information science research is needed to measure and track the spread and uptake of accurate and inaccurate information in both digital and physical environments, including the identification of sources of misinformation and disinformation (eg, policy briefs and crisis pregnancy centers); messaging content and strategies; the crossover of misinformation between digital and physical environments; and the association between exposure to misinformation and access to accurate health information, abortion policy, health inequities, and maternal outcomes. Social science and health communication research is needed to understand how different types of and exposures to misinformation and accurate information impact attitudes, beliefs, behavior, and health outcomes. Public health and behavioral science research is needed to develop and test interventions that prebunk or debunk misinformation, propagate accurate abortion information, increase health and media literacy, and better prepare people to navigate the information ecosystem. Additionally, systems- and policy-related research is needed to identify socioecological factors that moderate intervention efficacy, including individual-, community- and policy-level barriers to intervention implementation. Such research could inform the development of a more functional and resilient information ecosystem that is robust to legislation that attempts to curb information sharing. Research is also needed to determine how to support and equip health care providers with accurate information about abortion legislation in their state and how to effectively treat and counsel their patients within the confines of state laws. Generally, research is needed to develop a better understanding of the nature of the abortion infodemic, its contributing factors, and the efficacy of mitigation and resilience strategies.

Regardless of one’s view on abortion, the provision of medically accurate information is a basic human right, as underscored by the WHO [48]. Given the fast-evolving legislative landscape around the United States, the information ecosystem is also likely to be fast evolving, and social media platforms will accelerate the spread of misinformation if they fail to take appropriate action. We have learned from the COVID-19 pandemic that a coordinated research agenda is necessary when public health issues become politically charged and accompanied by a confusing information landscape. Such a research agenda must be supported via federal funding mechanisms that enable researchers to be agile in studying and effectively addressing evolving misinformation ecosystems. Importantly, research on the abortion infodemic is needed to inform policy, establish public health efforts that educate pregnant people on how to obtain safe reproductive care, maximize quality of reproductive care, and reduce health inequities in maternal outcomes. Marginalized communities may be the most negatively impacted by an abortion infodemic because they have higher rates of abortion [49,50], greater barriers to care [51,52], lower health literacy [53], less access to evidence-based health information [54], and less trust in health care providers resulting from a long legacy of systemic racism in health care [55]. The high US maternal mortality rate, for Black women in particular, is a known long-standing failure of the US health care system [22]. Without a data-driven approach to solutions, the abortion infodemic will exacerbate the impact of the *Roe v. Wade* reversal on maternal outcomes and health inequities for years to come.

## Abbreviations

SCOTUS: Supreme Court of the United States
WHO: World Health Organization
